# Severe pancytopenia caused by trifluridine/tipiracil in patients with metastatic colorectal cancer and an impaired renal function: A case report

**DOI:** 10.1002/ccr3.5544

**Published:** 2022-03-03

**Authors:** Masatsune Shibutani, Yuki Okazaki, Shinichiro Kashiwagi, Hisashi Nagahara, Tatsunari Fukuoka, Yasuhito Iseki, Kiyoshi Maeda, Kosei Hirakawa, Masaichi Ohira

**Affiliations:** ^1^ Department of Gastroenterological Surgery Osaka City University Graduate School of Medicine Osaka Japan; ^2^ Department of Breast and Endocrine Surgery Osaka City University Graduate School of Medicine Osaka Japan; ^3^ 13877 Department of Gastroenterological Surgery Osaka City General Hospital Osaka Japan

**Keywords:** colorectal cancer, febrile neutropenia, FTD/TPI, neutropenia, pancytopenia

## Abstract

Although the incidence of hematological toxicity due to Trifluridine/tipiracil (FTD/TPI) treatment is high, the incidence of severe adverse events has been reported to be relatively low. However, it should be noted that patients with renal impairment are prone to severe hematological adverse events.

## Graphical abstract

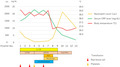

## 1 INTRODUCTION

Later‐line treatment has been shown to improve the survival of patients with metastatic colorectal cancer who are refractory to standard therapies, including fluoropyrimidines, oxaliplatin, irinotecan, anti‐vascular endothelial growth factor receptor monoclonal antibody, and anti‐epidermal growth factor receptor monoclonal antibody (RAS wild‐type only).^1^, ^2^ Trifluridine/tipiracil (FTD/TPI) is one of the anticancer drugs that is used in later‐line therapy for metastatic colorectal cancer. Although the incidence of hematological toxicity due to FTD/TPI treatment is high, it has been reported that the incidence of severe adverse events and discontinuation of treatment is relatively low and that most toxic effects can be managed by dose modification and/or delayed administration.^3^, ^4^ Furthermore, the tolerability has been confirmed, even in elderly patients.^4^, ^5^ However, the incidence of grade ≥3 hematological adverse events in patients with renal impairment was higher than that in patients with a normal renal function.^3^, ^6^ Therefore, great care should be taken when administering FTD/TPI to patients with an impaired renal function.

## 2 CASE PRESENTATION

An 83‐year‐old man with an impaired renal function who had undergone laparoscopic ileocecal resection, sigmoidectomy, and lymph node dissection for synchronous stage III ascending colon cancer, and stage III sigmoid colon cancer was found to have para‐aortic lymph node metastases and peritoneal dissemination 2 years after surgery. He received S‐1+oxaliplatin (SOX) + bevacizumab therapy as a first‐line treatment.

One year after the initiation of first‐line treatment, he was judged to have progressive disease due to the appearance of multiple pulmonary metastases. After the failure of first‐line treatment, he received 5‐fluorouracil + leucovorin + irinotecan (FOLFIRI) + Panitumumab therapy as second‐line treatment.

**FIGURE 1:**
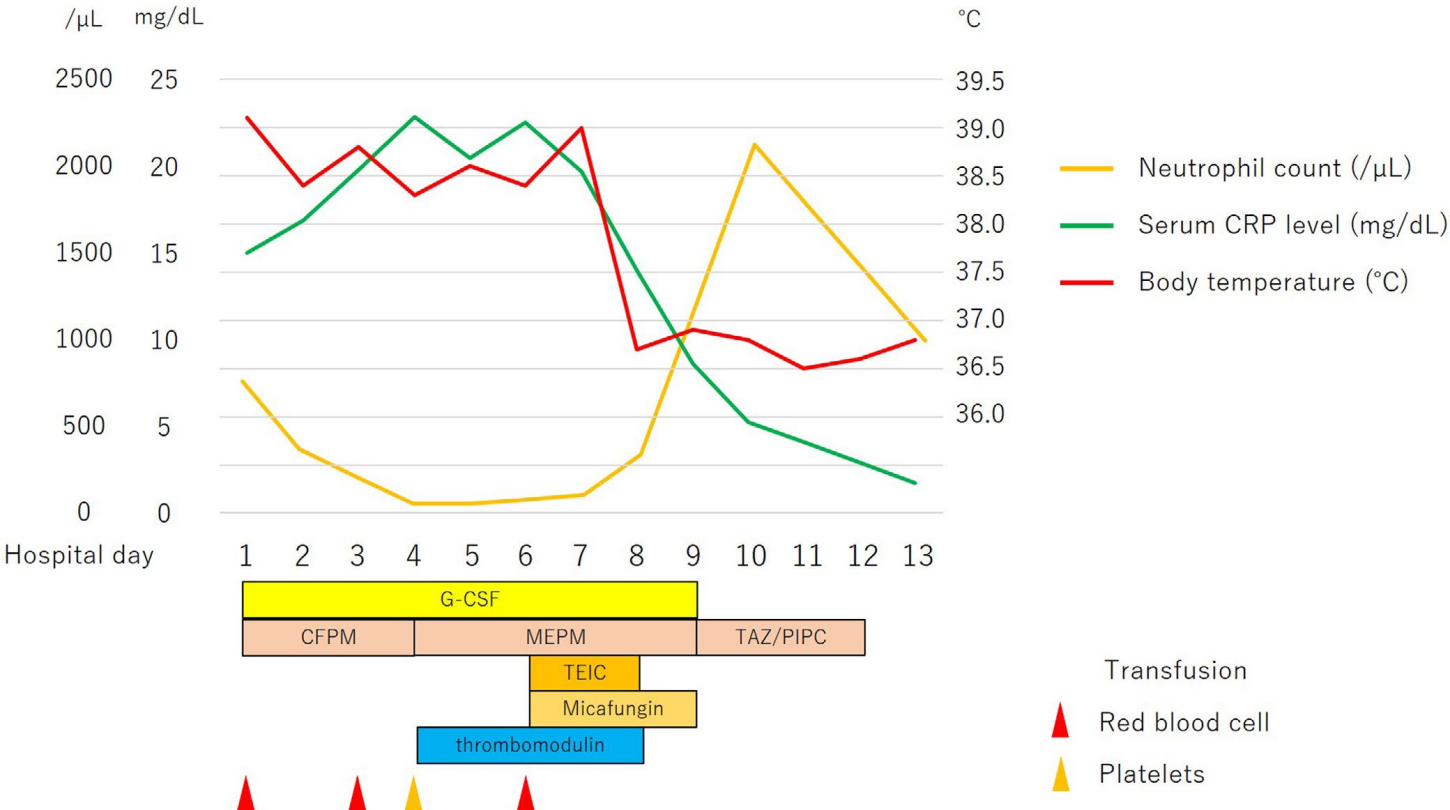
Clinical course. CFPM, cefepime dihydochloride hydrate; G‐CSF, granulocyte colony stimulating factor; MEPM, meropenem; TAZ/PIPC, Tazobactam/Piperacillin; TEIC, teicoplanin

Stable disease continued for 3 years with second‐line treatment, but eventually his multiple lung metastases increased. Therefore, he received FTD/TPI as third‐line treatment. Although the absolute neutrophil count and platelet count were sufficiently maintained before initiation of FTD/TPI treatment (Table 1), at 2 weeks after the initiation of FTD/TPI treatment, neutropenia (758/µl), and high fever were observed (Table 1), and he was admitted to hospital with a diagnosis of febrile neutropenia. The cause of high fever was a urinary tract infection. Despite the daily administration of granulocyte colony stimulating factor (G‐CSF), his neutrophil count dropped to <100/µl and sepsis developed on hospital Day 4 (Table 1). Furthermore, he was diagnosed with disseminated intravascular coagulation (DIC) based on the Japanese diagnostic criteria.^7^ The antibiotic agent was changed from cefepime dihydochloride hydrate to meropenem, and teicoplanin, antifungal drugs, and thrombomodulin were added. Furthermore, the central venous catheter was removed. In addition, due to the presence of pancytopenia with anemia and thrombocytopenia (Table 1), red blood cells and platelets were transfused. As the neutrophil count recovered, the infection improved on hospital Day 8 (Figure 1).

**TABLE 1:** Blood test results

	Before initiation of FTD/TPI treatment	Hospital day 1	Hospital day 4
White blood cells (/µl)	4800	1200	700
Hemoglobin (g/dl)	8.6	7.1	9.8
Platelets (/µl)	19.9 × 10^4^	6.1 × 10^4^	2.0 × 10^4^
Absolute neutrophil count (/µl)	1690	758	42
Total protein (g/dl)	5.7	5.8	5.5
Albumin (g/dl)	3.1	2.9	2.5
Total bilirubin (mg/dl)	0.2	0.4	0.9
AST (U/L)	20	19	18
ALT (U/L)	13	12	17
BUN (mg/dl)	16	31	32
Creatinine (mg/dl)	1.06	1.58	1.36
eGFR (ml/min/1.7)	51.39	33.09	38.99
C‐reactive protein (mg/dl)	1.88	14.99	22.83
Natrium (mmoL/L)	144	139	136
Kalium (mmoL/L)	4.3	4.4	4.3
Chloride (mmoL/L)	110	109	112
PT‐INR			1.39
FDP (µg/ml)			5.7

Abbreviations: ALT, alanine aminotransferase; AST, aspartate aminotransferase; BUN, blood urea nitrogen; eGFR, estimated glomerular filtration rate; FTD/TPI, trifluridine/tipiracil.

However, one month passed without being able to start the second course while his physical condition was unstable, and during that time, his multiple lung metastases increased.

## 3 DISCUSSION

Trifluridine/tipiracil is an oral anticancer drug containing trifluridine, which is an antineoplastic thymidine‐base nucleoside analog and tipiracil hydrochloride is a thymidine phosphorylase inhibitor.^8^, ^9^ Trifluridine is incorporated into DNA, causing DNA dysfunction,^9^, ^10^, ^11^ whereas tipiracil hydrochloride maintains the plasma concentration of trifluridine by inhibiting thymidine phosphorylase, which degrades trifluridine.^8^, ^9^ As tipiracil is primarily eliminated via renal excretion, the plasma concentration of trifluridine is likely to be high in patients with renal impairment,^12^ leading to an increased risk of adverse events.^6^ In previous studies, an association has been reported between a reduced renal function and an increased risk of adverse events.^3^, ^6^ However, there are still many unclear points about the correlation between the renal function and the plasma concentration of tipiracil and between the plasma concentration of tipiracil and the incidence of adverse events. Therefore, the criteria for dose reduction according to the renal function have not been established. As a result, in clinical practice, FTD/TPI is generally administered at a full dose, as long as the administration criteria are met. In our case, severe pancytopenia occurred, followed by febrile neutropenia and sepsis, which may have been due to the patient's decreased renal function. It may be necessary to adjust the dose of FTD/TPI based on the renal function, especially in elderly patients, because it is often difficult for elderly patients to recover once their condition becomes severe.

In our case, severe myelosuppression with a neutrophil count <100/µl lasted for one week; however, the time to the recovery of the bone marrow function was similar to the results in a previous study.^13^ Accordingly, it seems that the severity of myelosuppression and the duration of myelosuppression are not always correlated. Given that the increasing number of neutrophils due to the recovery of bone marrow function quickly improved the patient's infection, it is important to administer G‐CSF and control the infection until the bone marrow function is restored.

In our case, severe neutropenia occurred from the first course. Thus, it is necessary to pay attention to adverse events from the first course. Furthermore, in cases with severe neutropenia, the bone marrow function may be significantly reduced, and attention should be paid to the hemoglobin level and platelet count.

In our case, the disease progressed during the interruption of chemotherapy due to adverse events. Minimizing adverse events is important, as the disease may progress before recovery from adverse events. Biweekly administration has been reported as a method of preventing neutropenia.^14^ Furthermore, in a previous report, dose reduction from 35 mg/m^2^/twice daily to 20 mg/m^2^/twice daily was proposed for patients with an impaired renal function (glomerular filtration rate <30/ml/min) based on the predicted blood concentration for the prevention of neutropenia.^12^

## 4 CONCLUSION

Trifluridine/tipiracil treatment is associated with a high incidence of hematological toxicity, and may be more frequent in patients with an impaired renal function. Therefore, more caution is required when administering FTD/TPI treatment to patients with an impaired renal function.

## CONFLICT OF INTEREST

The authors declare no conflicts of interest in association with the present study.

## AUTHOR CONTRIBUTIONS

M. Shibutani drafted the manuscript. Y. Okazaki, S. Kashiwagi, H. Nagahara, T. Fukuoka, Y. Iseki, K. Maeda, K. Hirakawa, and M. Ohira critically reviewed the manuscript. All authors read and approved the final manuscript.

## CONSENT

Written informed consent was obtained from the patient for publication of this case report.

## Data Availability

The data that support the findings of this case report are available on request from the corresponding author.

## References

[1] Mayer RJ , Van Cutsem E , Falcone A , et al. Randomized trial of TAS‐102 for refractory metastatic colorectal cancer. N Engl J Med. 2015;372(20):1909‐1919. doi:10.1056/NEJMoa1414325 25970050

[2] Grothey A , Van Cutsem E , Sobrero A , et al. Regorafenib monotherapy for previously treated metastatic colorectal cancer (CORRECT): an international, multicentre, randomised, placebo‐controlled, phase 3 trial. Lancet. 2013;381:303‐312. doi:10.1016/S0140-6736(12)61900-X 23177514

[3] Falcone A , Ohtsu A , Van Cutsem E , et al. Integrated safety summary for trifluridine/tipiracil (TAS‐102). Anticancer Drugs. 2018;29:89‐96. doi:10.1097/CAD.0000000000000554 29176395

[4] Cicero G , Addeo R , De Luca R , et al. TAS‐102 in metastatic colorectal cancer (mCRC): efficacy, tolerability, and quality of life in heavily pretreated elderly patients: a real‐life study. Drugs Context. 2020;9:1‐8. doi:10.7573/dic.2020-6-3 PMC7505119 32994802

[5] Mayer RJ , Hochster HS , Cohen SJ , Winkler R , Makris L , Grothey A . Safety of trifluridine/tipiracil in an open‐label expanded‐access program in elderly and younger patients with metastatic colorectal cancer. Cancer Chemother Pharmacol. 2018;82:961‐969. doi:10.1007/s00280-018-3686-5 30350179

[6] Yoshino T , Uetake H , Funato Y , et al. Post‐marketing surveillance study of trifluridine/tipiracil in patients with metastatic colorectal cancer. Jpn J Clin Oncol. 2021;51:700‐706. doi:10.1093/jjco/hyaa243 33438718

[7] Gando S , Wada H , Asakura H , et al. Evaluation of new Japanese diagnostic criteria for disseminated intravascular coagulation in critically ill patients. Clin Appl Thromb Hemost. 2005;11:71‐76. doi:10.1177/107602960501100108 15678275

[8] Emura T , Suzuki N , Fujioka A , Ohshimo H , Fukushima M . Potentiation of the antitumor activity of alpha, alpha, alpha‐trifluorothymidine by the co‐administration of an inhibitor of thymidine phosphorylase at a suitable molar ratio in vivo. Int J Oncol. 2005;27:449‐455. 16010427

[9] Temmink OH , Emura T , de Bruin M , Fukushima M , Peters GJ . Therapeutic potential of the dual‐targeted TAS‐102 formulation in the treatment of gastrointestinal malignancies. Cancer Sci. 2007;98:779‐789. doi:10.1111/j.1349-7006.2007.00477.x 17441963 PMC11158373

[10] Tanaka N , Sakamoto K , Okabe H , et al. Repeated oral dosing of TAS‐102 confers high trifluridine incorporation into DNA and sustained antitumor activity in mouse models. Oncol Rep. 2014;32:2319‐2326. doi:10.3892/or.2014.3487 25230742 PMC4240496

[11] Sakamoto K , Yokogawa T , Ueno H , et al. Crucial roles of thymidine kinase 1 and deoxyUTPase in incorporating the antineoplastic nucleosides trifluridine and 2'‐deoxy‐5‐fluorouridine into DNA. Int J Oncol. 2015;46:2327‐2334. doi:10.3892/ijo.2015.2974 25901475 PMC4441292

[12] Saif MW , Becerra CR , Fakih MG , et al. A phase I, open‐label study evaluating the safety and pharmacokinetics of trifluridine/tipiracil in patients with advanced solid tumors and varying degrees of renal impairment. Cancer Chemother Pharmacol. 2021;88:485‐497. doi:10.1007/s00280-021-04308-z 34097100

[13] Yoshino T , Uetake H , Fujita N , et al. TAS‐102 safety in metastatic colorectal cancer: results from the first postmarketing surveillance study. Clin Colorectal Cancer. 2016;15:e205‐e211. doi:10.1016/j.clcc.2016.04.004 27324983

[14] Yoshida Y , Sakamoto R , Kajitani R , et al. Biweekly administration of TAS‐102 for neutropenia prevention in patients with colorectal cancer. Anticancer Res. 2018;38:4367‐4373. doi:10.21873/anticanres.12738 29970575

